# Similar Efficacy of Mesalazine in Adult and Older Adult Ulcerative Colitis Patients: Post Hoc Analysis of a Randomized Noninferiority Trial of 1600 mg vs 400 mg Tablets

**DOI:** 10.1093/ibd/izae123

**Published:** 2024-06-21

**Authors:** Ekaterina Safroneeva, Helen Thorne, Ortrud Gerstner, Raphaël Laoun

**Affiliations:** Tillotts Pharma AG, Rheinfelden, Switzerland; Institute of Social and Preventive Medicine, University of Bern, Bern, Switzerland; Tillotts Pharma AG, Rheinfelden, Switzerland; Tillotts Pharma AG, Rheinfelden, Switzerland; Tillotts Pharma AG, Rheinfelden, Switzerland

**Keywords:** randomized controlled clinical trial, elderly, ulcerative colitis, comorbidities, comedications, mesalazine, efficacy

## Abstract

**Background:**

The efficacy data on treatment in older adults are scarce, while the greatest increase in ulcerative colitis (UC) prevalence is observed in age groups of individuals 40 to 65 years of age and ≥65 years of age.

**Aim:**

We assessed the difference in rates of clinical and endoscopic response and remission in UC adults (≤60 years) and older adults (>60 years) treated with mesalazine.

**Methods:**

We performed a post hoc analysis of data from a phase 3 noninferiority trial of 817 UC patients treated with mesalazine for 8 and additional 26 weeks in a double-blind and open-label study, respectively. We used Wilcoxon rank sum or chi-square test to analyze differences between groups and multivariable logistic regression to determine the associations between endoscopic remission as outcome (Mayo endoscopic subscore [MES] = 0 or ≤1) and independent variables including disease duration, baseline MES, age, sex, comedications, and comorbidities.

**Results:**

Older adults had a longer disease duration, a higher number of comorbidities, concomitant medications, and higher baseline MES (2.38 ± 0.486 in older adults vs 2.26 ± 0.439 in adults; *P* = .008) compared with adults. We observed no difference in rates of combined clinical and endoscopic remission, clinical remission and response, and endoscopic remission and response at week 8 and 38 post-treatment. In addition to other well-known predictors of worse outcome, patients with ≥3 comedications were less likely to achieve an MES = 0 at week 8 and 38 and an MES ≤1 at week 38.

**Conclusions:**

We observed similar efficacy of mesalazine in adult and older adult UC patients. The increased comedication number rather than age may decrease effectiveness of UC medications, highlighting the importance of healthy aging.

## Graphical Abstract

**Graphical Abstract:**
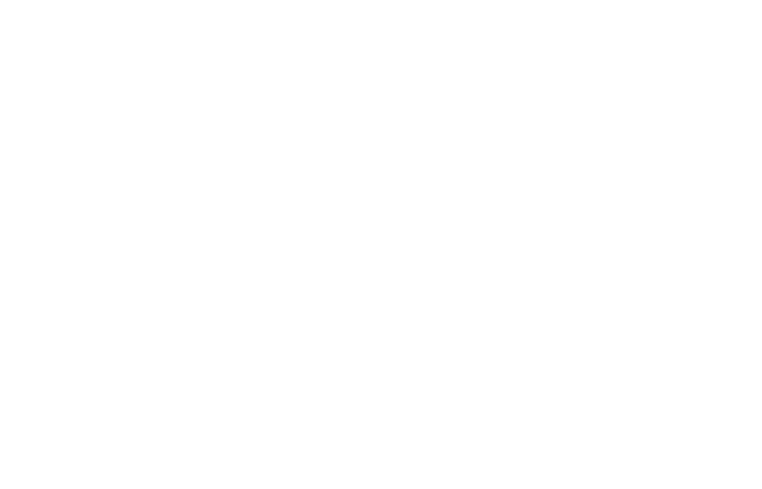

**Key Messages**

**What is already known?**

Available data on drug efficacy generally suggests no difference between elderly and adult populations for corticosteroids, thiopurines, anti-tumor necrosis factors, and vedolizumab; however, the data on efficacy of mesalazine in elderly populations are very limited.

**What is new here?**

In a post hoc analysis of the largest mesalazine clinical trial, we found that the efficacy of mesalazine is similar between adult and older adult ulcerative colitis patients.

**How can this study help patient care?**

Mesalazine is effective and safe in the older adult ulcerative colitis population.

## Introduction

As the global population is aging, the proportion of patients with chronic conditions is increasing. Ulcerative colitis (UC) incidence has a bimodal age distribution, with a peak in the second to fourth decades and a second smaller peak in the sixth to eighth decades of life. A recent Danish nationwide cohort reported that UC prevalence per 100 000 person-years increased from 235 (95% confidence interval [CI], 224-246) in 1995 to 832 (95% CI, 815-850) in 2016 in individuals of ≥65 years of age.^1^ The greatest increase in UC prevalence was observed among individuals 40 years of age or older.

Adults of older age (eg, >60 years) with inflammatory bowel disease (IBD) are underrepresented in clinical trials, as they are often systematically excluded. As a result, in Europe, no specific treatment algorithms have been developed for this age group.^2^ Therefore, European treatment guidelines currently do not distinguish between adults and older adults with respect to their recommendations.^3^

Although scarce, the currently available data on drug efficacy indicate that for most drugs, including corticosteroids, thiopurines, anti-tumor necrosis factors (anti-TNFs), and vedolizumab, there does not appear to be a difference between adult and older adult populations.^4-8^ Nevertheless, there are conflicting data.^9-11^ De Jong et al^10^ analyzed the data of multicenter prospective IBD registry and found that anti-TNF treatment failure rate, discontinuation of anti-TNF due to adverse events, and lack of anti-TNF effectiveness were higher in patients ≥60 years of age than in younger patients (subdistribution hazard ratios of 1.46, 1.52, and 1.11, respectively). In addition, a multicenter cohort study by Pugliese et al^11^ recently described a decreased rate of cumulative persistence on vedolizumab therapy in older patients (≥65 years of age) when compared with patients <65 years of age (51.4% vs 67.6%; log-rank test; *P* = .02). Although the authors could not match older adults and adult patients on previous anti-TNF exposure, older adults were less likely to be in clinical and steroid-free clinical remission. Multivariable analysis demonstrated that age <65 years (odds ratio, 1.72; 95% CI, 1.03-2.89; *P* = .038) was one of the predictors associated with steroid-free clinical remission at 24 months.

Given the methodological limitations associated with cohort studies, post hoc analyses of randomized controlled trials may offer a unique glimpse at the efficacy and safety of various medications in the older age population.^12^ Currently, studies investigating whether differences in efficacy between adults and the older-aged population exist for mesalazine treatment are lacking. A recent meta-analysis asked, “is it time to include older adults in inflammatory bowel disease trials?”—a very relevant question, as the proportion of the world’s population over 60 years of age between 2015 and 2050 is expected to nearly double from 12% to 22%.^13,14^

Therefore, to investigate if there is a difference in remission rates between adult and the older adult population with regard to mesalazine therapy, we carried out a post hoc analyses of multicenter, randomized, noninferiority trial of mesalazine (NCT01903252) in mild and moderate (Mayo score ≥5) adult UC patients. We compared the rates of clinical and endoscopic response and remission in adult (≤60 years of age) and older adult (>60 years of age) UC patients. We also assessed whether, among others, the presence of comorbidities and comedications is associated with endoscopic remission.

## Methods

The data from a multicenter, randomized, noninferiority trial of mesalazine (NCT01903252) were analyzed. This study was performed in accordance with the Declaration of Helsinki and the ICH Guidelines for Good Clinical Practice. Ethics committees at each study site approved the protocol, and all patients provided written informed consent. The study design and trial outcomes have been reported elsewhere.^15,16^ Briefly, during double-blind phase of the study, 817 adult patients (18 years of age or older) with mild and moderate UC (Mayo score ≥5 with rectal bleeding subscore ≥1, and Mayo endoscopic subscore [MES] ≥2 at baseline) were randomized to receive daily dose of 3.2 g oral mesalazine, given as either two 1600 mg tablets or eight 400 mg tablets for 8 weeks. The primary efficacy outcome was clinical and endoscopic remission at week 8. Subjects could continue with open-label treatment with the 1600 mg mesalazine tablet for up to 38 weeks, with different doses of mesalazine based on induction remission and response (reduction to 1.6 g/d in remitters, continuation with 3.2 g/d in responders, and dose intensification to 4.8 g/d in nonresponders).

Clinical and endoscopic remission was defined as a Mayo score of ≤2 points with no individual subscore >1 point. Clinical remission was defined as 0 points for both stool frequency and rectal bleeding on the partial Mayo score. Clinical and endoscopic response was defined as a decrease from baseline in the Mayo score of ≥3 points and >30% of the baseline score, with an accompanying decrease in the rectal bleeding subscore of ≥1 point or an absolute rectal bleeding subscore of 0 or 1, and clinical response was defined as a decrease in the partial Mayo score of ≥2 points and ≥30% from baseline, with a decrease in the rectal bleeding subscore of ≥1 point or absolute rectal subscore of 1 or 0.

Eligibility and endoscopic outcomes were evaluated by a single-blind central reader. Subjects were eligible to participate in this study based on considerations related to UC activity only. For the purposes of this study, we assessed outcomes that were prespecified in the clinical trial protocol. Complete data on 774 and 679 patients at week 8 of double-blind induction treatment and week 38 of open-label maintenance treatment, respectively, were available for the analysis.

Prescription and nonprescription medications received by the subject following screening were recorded in the case report form. Six or more months prior to randomization, 817 study subjects took up to 6 UC medications (261 with no UC medication, 468 with 1 UC medication, 59 with 2 UC medications, 29 with 3 or more medications) (**Table S1)**. Up to 3 months prior to randomization and during study participation, subjects were prohibited to take the following medications: rectal mesalamine, systemic or rectal corticosteroids, immunosuppressants, infliximab or other biologics, antibiotics, antidiarrheals, and nicotine patch. The exception was antibiotics to treat illnesses unrelated to UC. Subjects who required the initiation of prohibited medication (eg, corticosteroids) were considered treatment failures and were withdrawn from the study. To arrive at the number of comedications, all the therapies that were used during colonoscopy/sigmoidoscopy, as well as contraceptive medications, were excluded, while all the continuous medications were included. For the purposes of the induction and open-label maintenance treatment, other medications were considered overlapping if they were used for 2 and 30 days during the course of mesalazine treatment, respectively.

All adverse events were documented and classified according to the Medical Dictionary for Regulatory Activities (version 18.1; MedDRA).

### Data Handling and Statistical Analysis

Statistical analyses were performed using Stata the statistical program (version 16.1; StataCorp). Quantitative data distribution were analyzed using normal Q-Q plots. Demographic and clinical characteristics of adults with UC were summarized as frequencies and percentages or as medians, interquartile ranges, and ranges. The differences between groups were analyzed using the Wilcoxon rank sum or chi-square test with continuity correction. The pairwise relationship between comorbidities and comedications were analyzed with nonparametric correlations (Spearman’s ρ). The following definitions to interpret the Spearman’s correlation coefficients were applied: 0.0 to 0.3, weak; >0.3 to <0.7, moderate; and 0.7 or higher, strong relationship.

Logistic regression analysis in the overall population was performed with MES remission (defined as 0 or ≤1 according to blinded central reading) or Mayo clinical remission (defined as rectal bleeding and stool frequency subscores of 0) as the outcome and disease duration, MES at baseline, leukocyte concentration at baseline, Robarts Histopathologic Index at baseline, age (≤60 vs >60 years of age), sex, comedications at the time of the trial (none, ≤2, ≥3), comorbidities (none, 1-2, 3-5, >5), body mass index, disease extent prior to the trial, smoking status (current, ex-smoker, and never), and previous UC medications (within 6 months of randomization) at weeks 8 and 38 as predictors. Collinear variables (Spearman’s ρ correlation coefficient of >0.5( (**Figure S1**) were examined in separate models. No primary or secondary endpoints data were missing. If no data on comorbidities and comedications were available, we assumed that the subject had no comorbidities and comedications. A *P* value <.05 was considered statistically significant.

## Results

### Patient Characteristics

Of the 817 study participants, 128 subjects (49 female) were older adults (>60 years of age), while 689 (300 female) were ≤60 years of age. Older adult patients had longer disease duration, higher body mass index, and higher number of comorbidities recorded prior to the trial when compared with subjects ≤60 years of age (Table 1). Older adult patients also had higher baseline mean MES when compared with patients ≤60 years of age (2.38 ± 0.486 in older adults vs 2.26 ± 0.439 for adults ≤60 years of age; *P* = .008). When comedications and comorbidities were examined, older adult subjects were more likely to have vascular hypertensive disorders and corresponding use of antihypertensive medications (**Table S2**). No other differences with respect to baseline characteristics were observed.

**Table 1.** Baseline characteristics by age.

	18-60 y of age (n = 689)	>60 y of age (n = 128)	*P* value
Age, y	39.4 ± 10.94	66.7 ± 5.63	
Sex			
Female	300 (43.5)	49 (38.3)	.269
Male	389 (56.5)	79 (61.7)	
BMI, kg/m^2^	24.76 ± 4.502	27.31 ± 4.464	<.001^a^
Partial Mayo score	5.41 ± 1.135	5.28 ± 1.129	.253
Mayo score	7.67 ± 1.275	7.66 ± 1.377	.745
Urgency			
No	132 (19.2)	24 (18.8)	.914
Yes	557 (80.8)	104 (81.3)	
Time since diagnosis, mo	58.8 ± 72.81	100.7 ± 106.24	<.001^a^
Disease extent			
Proctitis (<15 cm)	11 (1.6)	0 (0.0)	.789
Proctosigmoiditis (15-25 cm from anal verge)	305 (44.3)	56 (43.8)	
Left sided colitis (to splenic flexure)	243 (35.3)	44 (34.4)	
Portion of transverse colon	21 (3.0)	4 (3.1)	
Pancolitis	102 (14.8)	23 (18.0)	
Other	5 (0.7)	1 (0.8)	
No endoscopy prior to visit 1	2 (0.3)	0 (0.0)	
Leukocyte count, ×10^9^/L	7.4 ± 2.2	7.8 ± 2.5	.184
Smoking status			
Current	52 (7.5)	6 (4.7)	.062
Ex-smoker	172 (25.0)	44 (34.4)	
Never	465 (67.5)	78 (60.9)	
Number of comorbidities^b^	0 (0-1, 0-16)	1 (0-4, 0-20)	<.001^a^
Number of comedications^b^	0 (0-1, 0-20)	1 (0-3, 0-12)	<.001^a^

Values are mean ± SD, n (%), or median (interquartile range, range).

Abbreviation: BMI, body mass index.

^a^ *P* value <.05 was considered statistically significant.

^b^The type of comorbidities and comedications are provided in **Supplementary Table S2**.

### Remission and Response Rates at Weeks 8 and 38 Post-Treatment With Mesalazine

We observed no difference in rates of combined clinical and endoscopic remission, clinical remission and response, and endoscopic remission and response at weeks 8 (n = 817) and 38 (n = 675) post-treatment with mesalazine (Table 2). Changes from baseline to end of treatment in Mayo score and patient-related outcomes are shown in **Table S3**.

**Table 2.** Remission, response, and urgency at weeks 8 and 38.

	18-60 y of age	>60 y of age	Difference (95% CI) (%) between age groups per time point, *P* value
Clinical and endoscopic remission (Mayo score ≤2 with no individual subscore >1)			
Week 8^a^	160 (23.2)	25 (19.5)	−3.7 (−11.7 to 4.3), .420
Week 38^b^	260 (45.5)	40 (38.8)	−6.6 (−17.5 to 4.2), .256
Clinical remission (stool frequency and rectal bleeding subscores of 0)			
Week 8^a^	174 (25.3)	33 (25.8)	0.5 (−8.2 to 9.2), .988
Week 38^b^	246 (43.0)	50 (48.5)	5.5 (−5.5 to 16.6), .350
Clinical response (decrease partial Mayo score of ≥2 points and ≥30 from week 0, with a decrease in the rectal bleeding subscore of ≥1 or absolute rectal bleeding subscore ≤1)			
Week 8^a^	446 (64.7)	80 (62.5)	2.2 (−11.8 to 7.3), .701
Week 38^b^	484 (84.6)	92 (89.3)	4.7 (−2.5 to 11.9), .275
Lack of urgency			
Week 8^a^	320 (47.7)	60 (49.2)	−1.5 (−11.6 to 8.6), .838
Week 38^b^	361 (65.3)	67 (65.7)	−0.4 (−11.0 to 10.2), 1.000
Endoscopic remission (Mayo endoscopic subscore ≤1)			
Week 8^a^	269 (39.0)	41 (32.0)	−7.0 (−15.9 to −1.9), .161
Week 38^b^	315 (55.1)	49 (47.6)	−7.5 (−18.0 to −3.0), .194
Endoscopic response (>1-point reduction in Mayo endoscopic subscore)			
Week 8^a^	333 (48.3)	56 (43.8)	−4.6 (−14.4 to 5.3), .392
Week 38^b^	351 (61.4)	65 (63.1)	1.7 (−9.0 to 12.5), .822

**Values are n (%) or n/n (%), unless otherwise indicated.**

**Abbreviation:** CI, confidence interval.

^a^For week 8, 689 patients 18-60 years of age and 128 patients >60 years of age were analyzed.

^b^For week 38, 572 patients 18-60 years of age and 103 patients >60 years of age were analyzed.

### Safety

The number of adverse events was significantly higher in older adult subjects when compared with subjects ≤60 years of age at both weeks 8 and 38; however, there were no significant differences in either severe or serious adverse events between the two groups reported at weeks 8 and 38 (Table 3) or timing when these events occurred (**Figure S2**).

**Table 3.** Summary of adverse events at week 8 and 38 (safety set).

	18-60 y of age (n = 689)	>60 y of age (n = 128)	Between-group difference (95% CI) (%)	*P* value
Week 8				
Any adverse event	162 (23.5)	45 (35.2)	11.6 (2.3 to 21.0)	.008^a^
Severe adverse events	18 (2.6)	2 (1.6)	−1.0 (−4.0 to 1.9)	.693
Drug-related adverse events	57 (8.3)	11 (8.6)	0.3 (−5.4 to 6.1)	1.000
Serious adverse events	11 (1.6)	3 (2.3)	0.7 (−2.5 to 4.0)	.820
Drug-related serious adverse events	3 (0.4)	0 (0.0)	0.4 (−1.4 to 0.5)	1.000
Adverse events leading to drug interruption	4 (0.6)	0 (0.0)	−0.6 (−1.6 to 0.4)	.861
Adverse events leading to drug discontinuation	36 (5.2)	12 (9.4)	4.2 (−1.6 to 9.9)	.103
Week 38				
Any adverse event	287 (41.7)	69 (53.9)	12.3 (2.4 to 22.1)	.014^a^
Severe adverse events	31 (4.5)	4 (3.1)	−1.4 (−5.2 to 2.5)	.640
Drug-related adverse events	120 (17.4)	19 (14.8)	−2.6 (−9.8 to 4.7)	.560
Serious adverse events	32 (4.6)	10 (7.8)	3.2 (−2.2 to 8.5)	.203
Drug-related serious adverse events	5 (0.7)	1 (0.8)	0.1 (−2.1 to 2.2)	1.000
Adverse events leading to drug interruption	6 (0.9)	0 (0.0)	−0.9 (−2.0 to 0.3)	.620
Adverse events leading to drug discontinuation	67 (9.7)	18 (14.1)	4.3 (−2.5 to 11.2)	.187

**Values are n (%), unless otherwise indicated.**

**Abbreviation:** CI, confidence interval.

^a^ *P* value <.05 was considered statistically significant.

### Correlation Between Comedication Use and Comorbidities

We analyzed data on 774 patients (330 female, 43.5 ± 14.3 years of age, median disease duration 3.00 years [interquartile range, 0.56-7.88 years]; 175 and 92 persons with 1-2 and ≥3 comedications, respectively) following 8 weeks of double-blind induction treatment. Similarly, we analyzed data on 679 patients (289 female, 43.7 ± 14.5 years of age, median disease duration 2.99 years [interquartile range, 0.57-7.62 years]; 150 and 85 persons with 1-2 and ≥3 comedications, respectively) that had colonoscopy/sigmoidoscopy at 38 weeks open-label maintenance phase. Among the users of 3 or more comedications, antihypertensives, antithrombotic agents, lipid-modifying agents, and drugs for peptic ulcer and gastroesophageal reflux disease were most used (data not shown).

### Predictors of Attaining Mucosal Healing

In a multivariable logistic regression with endoscopic remission as outcome, we analyzed known predictors associated with increased disease severity as well as the presence of comorbidities and comedications. As previously described, patients with a baseline MES of 3 and increased baseline leukocyte concentration were less likely to be in endoscopic remission irrespective of the endoscopic remission definition and time post-treatment (Table 4). In addition, patients with ≥3 comedications were less likely to be in endoscopic remission of 0 at weeks 8 and 38 and in endoscopic remission of ≤1 at week 38. Age >60 years was not a significant predictor in any of the models. Other predictors including disease extent, smoking status, body mass index, comorbidities, and previous UC medication use were not associated with the outcome (data not shown).

**Table 4.** ORs, 95% CIs, and *P* values for the multivariable logistic regression models with Mayo endoscopic remission at 8 and 38 weeks post-treatment with mesalazine as outcome.

	OR	95% CI	*P* value
Endoscopic remission of 0 at 8 wk (n = 80/774)			
>60 y of age	0.672	0.275-1.639	.382
Female	0.916	0.563-1.490	.724
Disease duration (per 10 y)	0.473	0.270-0.828	.009^a^
Comedications			
1-2	0.905	0.498-1.646	.744
≥3	0.203	0.047-0.869	.032^a^
Mayo endoscopic subscore of 3 at baseline	0.315	0.148-0.671	.003^a^
Log-transformed leukocytes at baseline	0.400	0.175-0.915	.030^a^
Endoscopic remission of ≤1 at 8 wk (n = 302/774)			
>60 y of age	1.041	0.660-1.641	.863
Female	1.099	0.809-1.493	.544
Disease duration (per 10 y)	0.811	0.634-1.037	.095
Comedications			
1-2	0.759	0.519-1.111	.156
≥3	0.622	0.368-1.049	.075
Mayo endoscopic subscore of 3 at baseline	0.288	0.196-0.424	<.001^a^
Log-transformed leukocytes at baseline	0.521	0.308-0.882	.015^a^
Endoscopic remission of 0 at 38 wk (n = 165/679)			
>60 y of age	0.586	0.313-1.097	.095
Female	1.368	0.949-1.971	.094
Disease duration (per 10 y)	0.668	0.472-0.946	.023^a^
Comedications			
1-2	0.825	0.519-1.309	.413
≥3	0.493	0.244-0.998	.049^a^
Mayo endoscopic subscore of 3 at baseline	0.508	0.317-0.814	.005^a^
Log-transformed leukocytes at baseline	0.386	0.204-0.729	.003^a^
Endoscopic remission of ≤1 at 38 wk (n = 357/679)			
>60 y of age	0.969	0.615-1.527	.894
Female	1.588	1.158-2.178	.004^a^
Disease duration (per 10 y)	0.900	0.704-1.151	.400
Comedications			
1-2	0.705	0.478-1.040	.078
≥3	0.560	0.337-0.930	.025^a^
Mayo endoscopic subscore of 3 at baseline	0.637	0.446-0.910	.013^a^
Log-transformed leukocytes at baseline	0.420	0.242-0.729	.002^a^

Abbreviations: CI, confidence interval; OR, odds ratio.

^a^ *P* value <.05 was considered statistically significant.

## Discussion

In this post hoc analysis of data from a randomized controlled clinical trial of adult UC patients treated with mesalazine, we observed no difference in rates of combined clinical and endoscopic remission, clinical remission or response, and endoscopic remission or response between adult (≤60 years of age) and older adult (>60 years of age) subjects at weeks 8 and 38 post-treatment. Importantly, no differences in serious adverse events were observed between the adult and older adult groups. These data emphasize the efficacy and safety of mesalazine in the older adult UC population. Given the relevant burden of comedications and comorbidities in older adult patients, we examined whether these factors might be predictors associated with endoscopic remission. We observed that patients with 3 or more comedications were less likely to be in endoscopic remission of 0 at weeks 8 and 38 and in endoscopic remission of ≤1 at week 38, indicating that relevant comorbidities requiring treatment, rather than the actual age, might impact mucosal healing rates. These findings highlight the importance of healthy aging in UC patients and are further discussed.

Oral mesalazine is the first line treatment for both induction and maintenance of remission in patients with mildly and moderately active UC.^3,17,18^ Although no 5-aminosalicylic acid trials reported age-specific subgroup analysis, given that mesalazine has been the mainstay first-line UC therapy and has an advantageous safety profile compared with other treatments for UC, it is used extensively in UC patients including older adults. These results are important, as mesalazine is one of the most frequently prescribed medications for the treatment of UC in older adults. For example, in a retrospective observational study in the United States, 5-aminosalicylic acid agents were the most frequently prescribed maintenance therapy in the older adult population, with 44% of older IBD patients being treated with this therapy.^7^ Although mesalazine contributes to pill burden in UC, the number of mesalazine tablets needed to be taken for an induction or maintenance dose has decreased over the past years, with daily 3 to 4 high-strength tablets providing an induction dose of 4.8 g/d. This, together with once daily dosage, simplifies the use of these therapies in daily clinical practice.^19^

Although adherence was assessed in this clinical study, it is likely that the data collected are not relatable to those coming from real world evidence, with 1-year mesalazine adherence rates as low as 24%.^20^ Adherence in the older adult population in general is likely a common problem and affected by variety of patient- and drug-related factors. For example, prevalence of nonadherence in older adults taking multiple medications (polypharmacy) prescribed for the same or multiple conditions has been reported to range from 6% to 55% in individuals of 65 years of age or older on multiple medications living at home, although the lack of uniformity with respect to measuring multiple medication adherence and definitions for polypharmacy pose challenges in the interpretation of these data.^21,22^ In the review by Zelko et al,^21^ nonadherence was associated with, among others, poor cognition and increasing number of drugs. Last, Gellad et al^23^ conducted a systematic review of studies examining the barriers to medication adherence for older adults ≥65 years of age in the United States. The authors found that, among others, comorbidity and the number of drugs taken had a mostly negative impact on adherence. Therefore, it appears that an increasing number of comorbidities and medications is negatively associated with adherence.

Nonadherence in real-world evidence studies, polypharmacy, and comorbidities, as well as other factors associated with aging like frailty, a relative immunodeficiency compared with younger patients, and altered drug metabolism, may, as observed in this study, impact efficacy/effectiveness of the medications, rather than chronological age itself.^24^ In this study, we observed that 3 or more comedications was an independent predictor for failing to achieve a MES≤1. Although the data on UC are scarce, in a retrospective observational study of adults visiting tertiary medical center outpatient clinic, 29.8% and 40.9% of the study population took ≥5 and 2 to 4 non-UC medications, respectively; major polypharmacy (≥5 non-UC medications) was significantly associated with an increased risk of UC flare (odds ratio, 4.00; 95% CI, 1.66-9.62) but not with the risk of therapy escalation, hospitalization, or surgery.^25^ The impact of adherence was not assessed in that study.

Mesalazine is considered to have a favorable risk profile, even when given in high doses (>4 g/d) and over long periods of time.^26^ In our analysis, we observed no difference in the number of severe and serious adverse events between the adult and the older adult populations at both the week 8 and 38 time points. Therefore, the current data in older adult patients provide no safety signals but should be interpreted with caution, given the post hoc nature of this analysis. Compared with other conventional therapies, mesalazine has fewer interactions.^27^ This makes this class of medications favorable for use in the older adult population, which is more likely to receive multiple medications when compared with younger individuals.

In our study, older adults had a longer disease duration when compared with adult subjects. When adjusting for disease duration in the logistic regression analysis, we observed no association between endoscopic remission, defined as MES≤1, and disease duration. However, subjects with longer disease duration were less likely to achieve endoscopic remission, defined as MES≤0. In a recently conducted systematic review, Ben-Horin et al^28^ observed no relationship between disease duration and clinical remission rates in UC patients recruited into the trials of advanced therapies. The authors did not examine the relationship between disease duration and endoscopic remission. Therefore, further studies are needed to address this question.

Our results should be interpreted with certain considerations in mind. This is a post hoc analysis of a randomized clinical trial that, among others, may be subject to bias. Hence, the results of this study should be interpreted with caution. No power calculations were carried out, as these do not indicate true power for detecting statistical significance, as post hoc power estimates are generally variable in the range of practical interest and can be very different from the true power.^29^ The group of older adult subjects may not be entirely representative of the general older adult UC patient population, as stringent inclusion criteria had to be met for entry into phase 3 of this noninferiority study, including the absence of any serious disease other than UC, which, in the opinion of the investigator, may have interfered with the subjects’ ability to fully participate in the study. In this study, we used 60 years of age as the cutoff agreed on by the European Crohn’s and Colitis Organization in a 2016 topical review on IBD in older adults.^30^ However, the systematic reviews by Vieujean et al^13^ and Kochar et al^31^ both used a cutoff of >65 years of age. While we observed a moderate correlation between the number of comedications and comorbidities, it appears that comorbidities requiring treatment are relevant, as only the number of comedications was associated with decreasing rates of endoscopic remission in the multivariable regression analysis. The association between the number of medications and comorbidities is not perfect, given that some comorbidities require multiple medications, whereas other multiple comorbidities, like allergic diseases, can be treated with 1 medication. In addition, the data on the relationship between endoscopic remission and comedication should be interpreted with caution, as they likely involve complex interplay among related concepts, such as frailty, increased comedication, decreased adherence, or increased pill burden, that may affect endoscopic remission rates in older patients. We did not collect data on frailty in patients recruited into this study. We observed no association between comorbidities linked to inflammaging and mucosal healing rates; larger studies are needed to examine this relationship. Although being the largest mesalazine study to date, the study was too small to be able to conduct meaningful comparisons between, among others, subjects with older adult–onset UC and non–older adult–onset UC, subjects above 75 and those 60 to 75 years of age, or newly diagnosed patients and those with long-standing UC. It was also not possible to assess whether adherence to mesalazine might have been impacted by comedication use and vice versa; real-world evidence would be necessary to answer this question.

Despite the limitations, our study had several strengths, particularly its multicenter randomized controlled trial design, 38-week duration, relatively large study size, rigorously collected safety data, accurate assessment of comorbidities and comedication use, and use of a central reader in evaluation of endoscopic findings.

## Conclusions

We observed similar rates of clinical and endoscopic response and remission in adult and older adult UC patients, with mildly and moderately active disease, treated with mesalazine for 8 and 38 weeks. In addition to other well-known factors, the increased number of comedications or associated factors, rather than the actual age, may be responsible for decreased efficacy of medications, including mesalazine, in older adult UC patients, highlighting the importance of healthy aging. The lower pill burden provided by the mesalazine 1600 mg may increase adherence and improve response to mesalazine medication in adults and the older adult population. Future studies should evaluate the impact of frailty, comedications, comorbidities, and pill burden on drug efficacy as well as on adherence.

## Supplementary data

Supplementary data is available at *Inflammatory Bowel Diseases* online.

izae123_suppl_Supplementary_Material

## Data Availability

Individual participant data will not be shared.
