# Supplementary material for: Similar Efficacy of Mesalazine in Adult and Older Adult Ulcerative Colitis Patients: Post Hoc Analysis of a Randomized Noninferiority Trial of 1600 mg vs 400 mg Tablets
Source: Inflamm Bowel Dis. 2024 Jun 21;31(4):975–82. doi: 10.1093/ibd/izae123 (PMC11985383; doi:10.1093/ibd/izae123)

**SUPPLEMENTARY TABLES AND FIGURES**

**Supplementary Table 1.** UC therapies taken 6 or more months prior to randomisation for 817 subjects.^+^

|  | **≤60 years (n=576)** | **>60 years**  **(n=108)** | **Total**  **(n=684)** |
| --- | --- | --- | --- |
| Corticosteroids acting locally | 30 | 7 | 37 |
| Aminosalicylic acid and similar agents | 452 | 91 | 543 |
| Glucocorticoids oral | 11 | 5 | 16 |
| Other immunosuppressants | 5 | 1 | 6 |
| Other drugs for peptic ulcer and gastro-oesophageal reflux disease | 20 | 1 | 21 |
| Others | 58 | 3 | 61 |

^+^Some subjects were taking more than one therapeutic agent.

**Supplementary Table 2.** Top 10 co-morbidities and comedications in the adult (≤ 60 years) and older adult (>60 years) populations.

|  | **≤ 60 years** | | **> 60 years** | | **Total** | |
| --- | --- | --- | --- | --- | --- | --- |
| **Co-morbidities** | **Freq.** | **%** | **Freq.** | **%** | **Freq.** | **%** |
| Vascular hypertensive disorders necrotizing enterocolitis | 48 | 6 | 48 | 16 | 96 | 9 |
| Gastritis (excl infective) | 41 | 6 | 8 | 3 | 49 | 5 |
| Anaemias NEC | 42 | 6 | 6 | 2 | 48 | 5 |
| Cholecystitis and cholelithiasis | 28 | 4 | 6 | 2 | 34 | 3 |
| Gastrointestinal atonic and hypomotility | 24 | 3 | 10 | 3 | 34 | 3 |
| Bronchospasm and obstruction | 20 | 3 | 8 | 3 | 28 | 3 |
| Diabetes mellitus (including subtypes) | 14 | 2 | 10 | 3 | 24 | 2 |
| Acute and chronic pancreatitis | 20 | 3 | 2 | 1 | 22 | 2 |
| Haemorrhoids and gastrointestinal varices | 18 | 2 | 4 | 1 | 22 | 2 |
| Ischaemic coronary artery disorders | 9 | 1 | 8 | 3 | 17 | 2 |
| Other conditions | 478 | 64 | 189 | 63 | 667 | 66 |
| **ATC 3rd level pharmacological subgroup** | **Freq.** | **%** | **Freq.** | **%** | **Freq.** | **%** |
| Antihypertensives* | 72 | 17 | 86 | 32 | 158 | 23 |
| Drugs for peptic ulcer and gastro-oesophageal reflux disease | 25 | 6 | 14 | 5 | 39 | 6 |
| Iron preparations | 31 | 7 | 7 | 3 | 38 | 6 |
| Lipid modifying agents | 15 | 4 | 15 | 6 | 30 | 4 |
| Other analgesics and antipyretics | 21 | 5 | 9 | 3 | 30 | 4 |
| Blood glucose lowering drugs and insulins | 21 | 5 | 5 | 2 | 26 | 4 |
| Anti-thrombotic agents | 10 | 2 | 14 | 5 | 24 | 4 |
| Thyroid preparations (hypothyrosis) | 8 | 2 | 10 | 4 | 18 | 3 |
| Adrenergics, inhalants | 12 | 3 | 2 | 1 | 14 | 2 |
| Antihistamines for systemic use | 12 | 3 | 2 | 1 | 14 | 2 |
| Other medications | 189 | 45 | 101 | 38 | 290 | 43 |

*from different ATC groups (C2, C3, C7, C8, C9)

**Supplementary Table 3.** Change from baseline to end of treatment in Mayo clinic score and patient-related outcomes.

|  | **18 to 60 years of age**  **n=689** | **>60**  **years of age**  **n=128** | **Difference** | **95% CI** | **P-value** |
| --- | --- | --- | --- | --- | --- |
| Change in total Mayo clinic score     Week 8     Week 38 | -3.17  -4.53 | -3.04  -4.44 | -0.13  -0.09 | (-0.64, 0.38)  (-0.59, 0.41) | 0.608  0.716 |
| Change in Mayo clinic endoscopic score     Week 8     Week 38 | -0.62  -0.93 | -0.50  -0.80 | -0.12  -0.13 | (-0.29, 0.05)  (-0.34, 0.08) | 0.174  0.222 |
| Change in stool frequency     Week 8     Week 38 | -1.62  -2.26 | -1.56  -2.28 | -0.06  0.02 | (-0.52, 0.40)  (-0.39, 0.42) | 0.798  0.941 |
| Change in rectal bleeding score     Week 8     Week 38 | -0.87  -1.14 | -0.92  -1.19 | 0.05  0.05 | (-0.08, 0.19)  (-0.07, 0.17) | 0.437  0.389 |

All analyses are based on a mixed model for repeated measures and are adjusted for the baseline value of the respective outcome.

**FiGURES**

**Supplementary Figure 1.** Scatter plots of comorbidities vs. comedications used over 8-week (**A**) and 38-week (**B**) period.


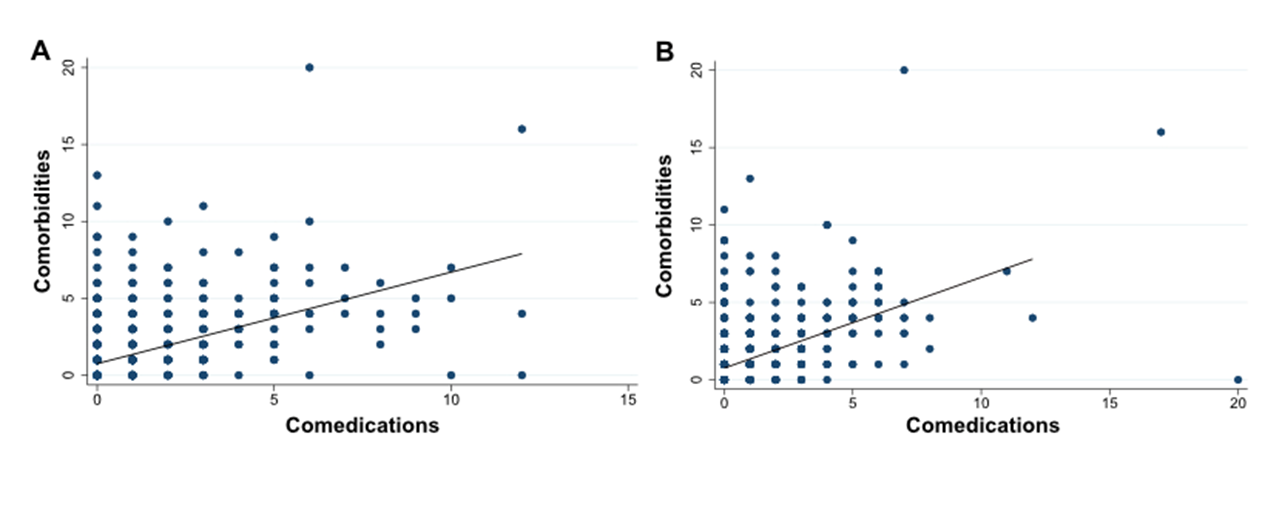


**Supplementary Figure 2.** Time to first treatment-emergent adverse event (safety set).


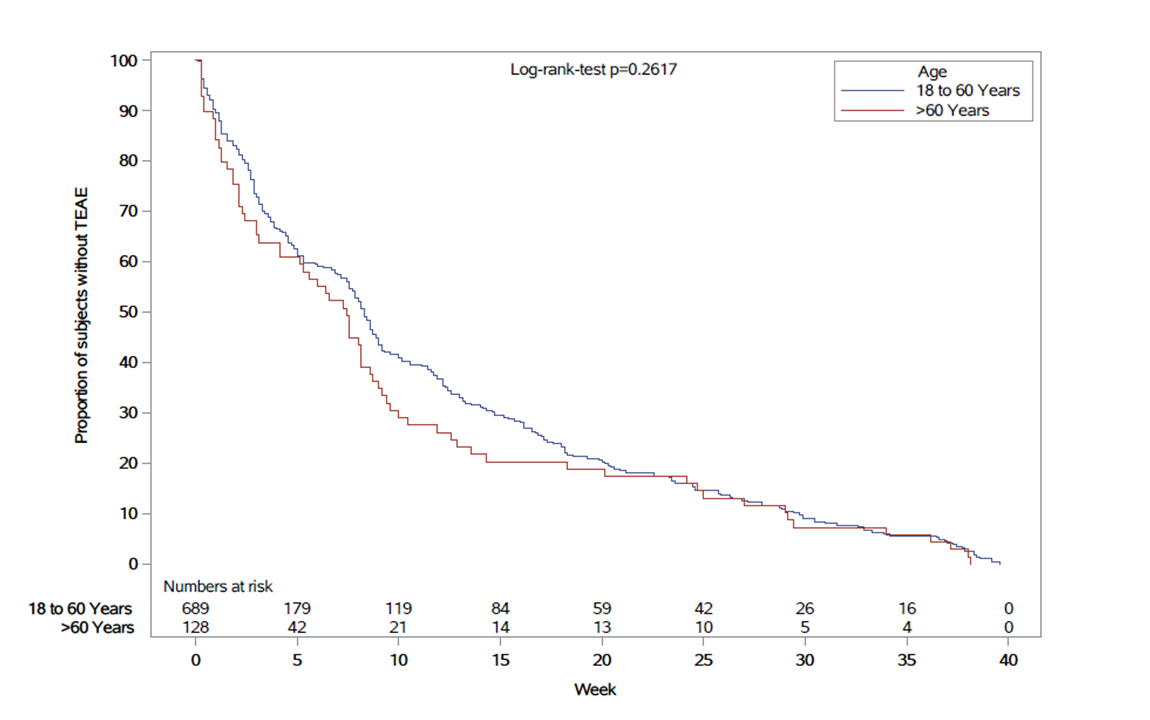

Supplement: izae123_suppl_Supplementary_Material [file izae123_suppl_supplementary_material.docx]
